# Perceived risk profile and treatment optimization in heart failure: an analysis from BIOlogy Study to TAilored Treatment in chronic heart failure

**DOI:** 10.1002/clc.23576

**Published:** 2021-05-07

**Authors:** Masatake Kobayashi, Adriaan A. Voors, Wouter Ouwerkerk, Kevin Duarte, Nicolas Girerd, Patrick Rossignol, Marco Metra, Chim C. Lang, Leong L. Ng, Gerasimos Filippatos, Kenneth Dickstein, Dirk J. van Veldhuisen, Faiez Zannad, João Pedro Ferreira

**Affiliations:** ^1^ Université de Lorraine, INSERM, Centre d'Investigations Cliniques Plurithématique 1433, Inserm U1116, CHRU de Nancy and F‐CRIN INI‐CRCT Nancy France; ^2^ Department of Cardiology University of Groningen, University Medical Center Groningen Groningen Netherlands; ^3^ National Heart Centre Singapore, Hospital Drive Singapore; ^4^ Department of Dermatology Amsterdam UMC, Amsterdam Infection & Immunity Institute, University of Amsterdam Amsterdam Netherlands; ^5^ Cardiology. University and Civil hospitals of Brescia Brescia Italy; ^6^ Division of Molecular and Clinical Medicine School of Medicine, University of Dundee, Ninewells Hospital & Medical School Dundee UK; ^7^ Department of Cardiovascular Sciences University of Leicester, NIHR Leicester Biomedical Research Centre, Glenfield Hospital Leicester UK; ^8^ Athens University Hospital Athens Greece; ^9^ Department of Internal Medicine University of Bergen Bergen Norway; ^10^ Department of Cardiology Stavanger University Hospital Stavanger Norway

**Keywords:** ACE‐inhibitor, adverse effects, ARB, beta‐blocker, treatment up‐titration

## Abstract

**Background:**

Achieving target doses of angiotensin‐converting‐enzyme inhibitor/angiotensin‐receptor blockers (ACEi/ARB) and beta‐blockers in heart failure with reduced ejection fraction (HFrEF) is often underperformed. In BIOlogy Study to TAilored Treatment in chronic heart failure (BIOSTAT‐CHF) study, many patients were not up‐titrated for which no clear reason was reported. Therefore, we hypothesized that perceived‐risk profile might influence treatment optimization.

**Methods:**

We studied 2100 patients with HFrEF (LVEF≤40%) to compare the clinical characteristics and adverse events associated with treatment up‐titration (after a 3‐month titration protocol) between; a) patients not reaching target doses for unclear reason; b) patients not reaching target doses due to symptoms and/or side effects; c) patients reaching target doses.

**Results:**

For ACEi/ARB, (a), (b) and (c) was observed in 51.3%, 25.9% and 22.7% of patients, respectively. For beta‐blockers, (a), (b) and (c) was observed in 67.5%, 20.2% and 12.3% of patients, respectively. By multinomial logistic regression analysis for ACEi/ARB, patients in group (a) and (b) had lower blood pressure and poorer renal function, and patients in group (a) were older and had lower ejection fraction. For beta‐blockers, patients in group (a) and (b) had more severe congestion and lower heart rate. At 9 months, adverse events (i.e., hypotension, bradycardia, renal impairment, and hyperkalemia) occurred similarly among the three groups.

**Conclusions:**

Patients in whom clinicians did not give a reason why up‐titration was missed were older and had more co‐morbidities. Patients in whom up‐titration was achieved did not have excess adverse events. However, from these observational findings, the pattern of subsequent adverse events among patients in whom up‐titration was missed cannot be determined.

## 1 INTRODUCTION

Up‐titration of angiotensin converting enzyme inhibitor/angiotensin receptor blockers (ACEi/ARB) and beta‐blockers to target doses reduces morbidity and mortality in heart failure with reduced ejection fraction (HFrEF).^1^, ^2^, ^3^, ^4^, ^5^, ^6^, ^7^ Nonetheless, HF medications target doses are often not achieved in clinical practice.^8^, ^9^, ^10^, ^11^

A systems BIOlogy Study to TAilored Treatment in chronic heart failure (BIOSTAT‐CHF) included patients on suboptimal HFrEF therapy in whom clinicians were encouraged (by protocol) to up‐titrate to target doses ACEi/ARBs and beta‐blockers during first 3‐months after inclusion.^12^ In a previous BIOSTAT‐CHF report, it was shown that patients treated with less than 50% of recommended ACEi/ARB and/or beta‐blocker doses had a poorer prognosis.^13^

Physicians participating in BIOSTAT‐CHF were specifically instructed to record the reasons for not achieving the recommended target doses. Intriguingly, in most patients, no clear reason was provided for not reaching target doses. In the present study, we hypothesized that, in such cases, the 'unspecified reasons' might be related to perceived but unreported higher patient‐risk profile, or to concern about the risk of expected patients' intolerance.^14^ Characterization and treatment‐related adverse events of these patients may help improve guideline‐recommended treatment optimization.

To this aim, we compared baseline characteristics and adverse events associated with attempts of treatment up‐titration among patients not reaching target doses for unspecified reason, those not reaching target doses due to symptoms and/or side effects, and those reaching target doses.

## 2 METHODS

### 2.1 Patient population

The description of the BIOSTAT‐CHF cohort has been previously published.^12^, ^13^ In brief, BIOSTAT‐CHF was an investigator‐driven multi‐center clinical study consisting of 2516 patients from 69 centers in 11 European countries with symptoms of HF, which was confirmed by left ventricular ejection fraction (LVEF) ≤40% and/or brain natriuretic peptide >400 pg/ml or N‐terminal pro BNP (NT‐proBNP) >2000 pg/ml and treatment of furosemide. Patients were receiving <50% of the guideline‐recommended target doses of at least one of ACEi/ARBs and beta‐blockers at the time of inclusion (visit 1). Patients underwent a 3‐month up‐titration period when the treating physicians were encouraged to initiate or up‐titrate ACEi/ARB and beta‐blockers to target doses.^15^ For the following 6 months, no further medication changes were mandated unless clinically indicated, and the 9‐month visit (visit 2) was performed.

For this analysis, we included patients with a LVEF≤40% who survived at the end of first 3‐month up‐titration period as previously published.^13^ Patients were considered successfully up‐titrated when patients achieved guideline‐recommended target dose after the 3‐months up‐titration period. According to recorded reasons for not reaching target doses in the case report form (CRF), patients were divided into three groups as previously shown^13^: (a) those in whom clinicians did not report a reason for not reaching target doses, (b) those who did not reach target doses because they experienced symptom, side effects or non‐cardiac organ dysfunction, and (c) those who reached target doses.

Ethics Board approval was obtained, and all participants signed written informed consent prior to entry into the study.

### 2.2 Adverse events associated with ACEi/ARB or beta‐blockers and high‐risk subgroups

The incidence of adverse events was assessed at the 9‐month visit (visit 2), which was pre‐specified for ACEi/ARB and beta‐blockers, and included the occurrence of hypotension (systolic blood pressure < 90 mmHg, hyperkalemia (potassium concentration of >5.0 and > 5.5 mmoL/L of potassium), renal impairment [estimated glomerular filtration rate (eGFR, as calculated by the Chronic Kidney Disease Epidemiology Collaboration formula^16^) <30 ml/min/1.73m^2^] and bradycardia (heart rate < 50 bpm).

We also considered “high‐risk” subgroups as previously reported,^17^, ^18^, ^19^, ^20^, ^21^, ^22^ as patient subpopulations that are more likely to experience adverse events. These subgroups included: those with an age ≥ 75 years, a body mass index (BMI) ≤25 kg/m^2^, a systolic blood pressure (SBP) ≤120 mmHg, a heart rate ≤ 70 bpm, diabetes, New York Heart Association (NYHA) class of III or IV, a LVEF ≤30%, Kansas City Cardiomyopathy Questionnaire score (KCCQ score) ≤ median (60), and an eGFR ≤60 mL/min/1.73m^2^.

### 2.3 Statistical analysis

Categorical variables are described as frequencies (percentages) and continuous variables are described as means ± standard deviation or median (25% and 75%), depending on their distribution. Comparisons of demographic, clinical and biological parameters among patients for whom reason was not reported, those experiencing symptoms/side effects and those reaching target doses were conducted using χ^2^ tests for categorical variables and ANOVA or Kruskal‐Wallis test for continuous variables.

Propensity score methods (i.e., matching, inverse probability weighting and propensity score adjustment) has been reported to be not necessarily superior to covariate adjustment.^23^ We thus report results of covariate adjustment as the primary analysis. Considering patients treated with target doses to be a reference group, clinical determinants of those on suboptimal doses for unspecified reason or those due to symptoms/side effects were selected in multinomial logistic regression analysis with backward selection. All available covariates with a small proportion of missing values (<10%) were included in the models for ACEi/ARB and beta‐blockers, and no multiple imputation was performed. Potential confounders selected herein were adjusted to compare incidences of adverse events associated with ACEi/ARB and beta‐blocker among patients for whom reason was not reported, those experiencing symptoms/side effects, and those reaching target doses.

All analyses were performed using R (R Development Core Team, Vienna, Austria). p value< .05 was considered statistically significant.

## 3 RESULTS

### 3.1 Baseline characteristics

Among a total of 2100 patients included in the present study, 75.7% were male, the mean age was 68.0 ± 12.0 years, the mean LVEF was 28.6 ± 7.5% and the mean eGFR was 61.7 ± 24.1 ml/min/1.73m^2^.

**FIGURE 1:**
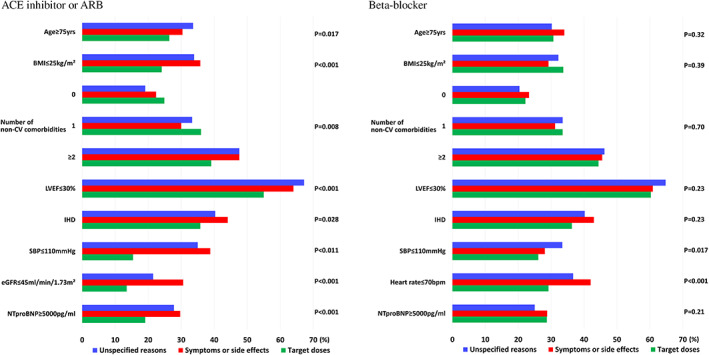
Main clinical characteristics. Non‐cardiovascular comorbidities were defined as any of the following: diabetes mellitus, thyroid dysfunction, anemia, chronic kidney disease (estimated glomerular filtration rate < 60 ml/min/1.73m^2^), chronic obstructive pulmonary disease, current smoking and current cancer. ACEi, angiotensin converting enzyme inhibitor; ARB, angiotensin receptor blocker; BMI, body mass index; CV, cardiovascular; LVEF, left ventricular ejection fraction; IHD, ischemic heart disease; SBP, systolic blood pressure; eGFR, estimated glomerular filtration rate; NT‐proBNP, N‐terminal pro‐B‐type natriuretic peptide

The baseline characteristics of patients in whom (a) no reason was reported for not up‐titrating ACEi/ARB or beta‐blockers versus (b) those experiencing symptoms/side effects versus (c) those reaching target doses are shown in the Table 1 and Figure 1. For ACEi/ARB, the distribution according to the aforementioned categories was: (a) 51.3% (*N* = 1061), (b) 25.9% (*N* = 536) and (c) 22.7% (*N* = 470). Patients reaching target doses had higher BMI, less frequent ischemic heart disease, less prior HF hospitalization, less congestive signs, and symptoms, higher LVEF, SBP and better renal function (all p values< .05).

**TABLE 1:** Baseline characteristicss

	ACE inhibitor/ARB				Beta‐blocker			
	Unspecified reasons (*N* = 1061)	Symptoms or side effects (*N* = 536)	Target doses (*N* = 470)	p value	Unspecified reasons (*N* = 1405)	Symptoms or side effects (*N* = 420)	Target doses (*N* = 257)	p value
Age, yrs	67.6 ± 12.0	67.4 ± 12.3	66.2 ± 11.6	.08	67.0 ± 11.9	68.4 ± 11.5	66.6 ± 12.9	0.11
Male, *N* (%)	258 (24.3%)	137 (25.6%)	109 (23.2%)	0.68	326 (23.2%)	109 (26.0%)	71 (27.6%)	0.21
Body mass index, kg/m^2^	27.7 ± 5.3	27.3 ± 5.0	29.4 ± 6.2	**<.0001**	28.0 ± 5.6	28.1 ± 5.3	27.9 ± 5.7	0.66
Medical history, *N* (%)								
Hypertension	618 (58.2%)	298 (55.6%)	335 (71.3%)	**<.0001**	842 (59.9%)	266 (63.3%)	159 (61.9%)	0.43
Diabetes mellitus	339 (32.0%)	149 (27.8%)	175 (37.2%)	**.006**	466 (33.2%)	122 (29.0%)	83 (32.3%)	0.28
Ischemic heart disease	427 (40.3%)	236 (44.1%)	168 (35.8%)	**.03**	565 (40.2%)	180 (43.0%)	93 (36.3%)	0.23
Atrial fibrillation	455 (42.9%)	242 (45.1%)	190 (40.4%)	0.32	594 (42.3%)	173 (41.2%)	129 (50.2%)	**.043**
Prior HF hospitalization	344 (32.4%)	189 (35.3%)	125 (26.6%)	**.01**	444 (31.6%)	129 (30.7%)	92 (35.8%)	0.35
COPD	186 (17.5%)	87 (16.2%)	67 (14.3%)	0.28	238 (16.9%)	68 (16.2%)	36 (14.0%)	0.50
Peripheral artery disease	104 (9.8%)	67 (12.5%)	40 (8.5%)	.09	159 (11.3%)	35 (8.3%)	19 (7.4%)	.06
Precipitating factors, *N* (%)								
Acute coronary syndrome	60 (5.8%)	20 (3.8%)	18 (3.9%)	0.14	66 (4.8%)	27 (6.6%)	7 (2.8%)	.09
Atrial fibrillation	219 (20.8%)	121 (22.8%)	92 (19.7%)	0.47	294 (21.0%)	80 (19.4%)	62 (24.5%)	0.29
Renal failure	90 (8.5%)	70 (13.1%)	21 (4.5%)	**<.0001**	119 (8.5%)	46 (11.1%)	20 (7.8%)	0.22
Clinical examinations								
NYHA III + IV, *N* (%)	622 (60.0%)	338 (65.0%)	254 (55.2%)	**.007**	838 (61.0%)	246 (60.3%)	143 (57.0%)	0.49
Leg edema, *N* (%)	513 (57.8%)	247 (56.3%)	212 (56.1%)	0.79	671 (57.4%)	188 (54.0%)	122 (61.3%)	0.24
Hepatomegaly, *N* (%)	158 (14.9%)	71 (13.3%)	60 (12.8%)	0.47	197 (14.1%)	58 (13.8%)	33 (12.8%)	0.88
Systolic BP, mmHg	121.7 ± 19.8	121.9 ± 21.7	132.5 ± 21.8	**<.0001**	123.9 ± 21.7	124.2 ± 20.7	125.5 ± 20.0	0.41
Heart rate, bpm	79.7 ± 19.1	80.5 ± 19.5	79.7 ± 20.6	0.55	79.4 ± 18.4	77.7 ± 19.9	86.0 ± 23.0	**<.0001**
LVEF, %	28.1 ± 7.4	28.5 ± 7.7	29.7 ± 7.3	**.0005**	28.4 ± 7.4	28.9 ± 7.7	28.8 ± 7.5	0.32
Medications, *N* (%)								
ACEi/ARB	769 (72.5%)	381 (71.1%)	383 (81.5%)	**.0002**	1057 (75.2%)	307 (73.1%)	190 (73.9%)	0.65
Beta‐blocker	898 (84.6%)	445 (83.0%)	409 (87.0%)	0.21	1169 (83.2%)	353 (84.0%)	243 (94.6%)	**<.0001**
MRA	607 (57.2%)	299 (55.8%)	242 (51.5%)	0.11	814 (57.9%)	216 (51.4%)	124 (48.2%)	**.003**
Loop diuretics	1058 (99.7%)	534 (99.6%)	467 (99.4%)	0.59	1397 (99.4%)	420 (100.0%)	256 (99.6%)	0.29
Digitalis	208 (19.6%)	109 (20.3%)	80 (17.0%)	0.37	277 (19.7%)	80 (19.0%)	43 (16.7%)	0.53
Laboratory findings								
Hemoglobin, g/dl	13.3 ± 1.8	13.2 ± 2.0	13.7 ± 1.8	**.0002**	13.4 ± 1.9	13.3 ± 1.8	13.4 ± 1.8	0.79
Sodium, mmol/l	139.0 ± 3.9	139.1 ± 3.8	139.9 ± 3.7	**<.0001**	139.2 ± 3.9	139.2 ± 3.8	139.4 ± 3.6	0.98
Potassium, mmol/l	4.3 ± 0.5	4.3 ± 0.6	4.2 ± 0.5	0.45	4.3 ± 0.6	4.3 ± 0.6	4.3 ± 0.5	0.28
Blood urea nitrogen, mg/dl	42.8 ± 33.1	45.5 ± 33.9	32.5 ± 28.9	**<.0001**	41.9 ± 32.2	42.7 ± 36.7	35.0 ± 26.4	**.003**
eGFR, ml/min/1.73m^2^	63.4 ± 24.3	59.4 ± 24.2	67.7 ± 22.8	**<.0001**	63.3 ± 24.0	61.6 ± 22.7	66.0 ± 25.9	0.14
NT‐proBNP, pg/ml	2566 (1098–5802)	2967 (1336–5805)	1909 (793–4068)	**<.0001**	2468 (1080–4999)	2578 (1110–5793)	2558 (1180–5620)	0.38

*Note*: Values are Mean ± SD, *n* (%) or median (25–75%).

Bold values if *p*‐value < 0.05.

Abbreviations: ACEi, angiotensin converting enzyme inhibitor; ARB, angiotensin receptor blocker; BP, blood pressure; COPD, chronic obstructive pulmonary disease; LVEF, left ventricular ejection fraction; MRA, mineralocorticoid receptor antagonist; eGFR, estimated glomerular filtration rate; NT‐proBNP, N‐terminal pro‐B‐type natriuretic peptide.

For beta‐blockers the distribution was: (a) 67.5% (*N* = 1405), (b) 20.2% (*N* = 420) and (c) 12.3% (*N* = 257). Patients reaching target doses had more frequent atrial fibrillation, and higher heart rate (all p values< .05).

Detailed information about no up‐titration of ACEi/ARB or beta‐blockers due to symptom, side effects or non‐cardiac organ dysfunction is shown in Supplementary Table 1.

### 3.2 Association of baseline characteristics with patients not reaching target doses for unspecified reasons or those not reaching target doses due to symptoms/side effects

In multinomial logistic regression model for ACEi/ARB, lower BMI, ischemic heart disease, more severe congestion, lower SBP and poorer renal function were associated with less frequent up‐titration to target doses (in both groups (a) and (b)) that is, without and with reason provided); whereas those from Northern European centers and those with diabetes were more likely to reach target doses. In addition, older age and lower ejection fraction were associated with less frequent up‐titration in the group without specified reason (all p values< .05). Table 2.

**TABLE 2:** Multinomial logistic regression model for patients not reaching target doses for unspecified reasons or those not reaching target doses due to symptoms and/or side‐effects

ACEi/ARB model	Unspecified reasons		Symptoms or side effects	
	OR (95% CI)	p value	OR (95% CI)	p value
European geographical area				
Central countries	(reference)		(reference)	
North countries	0.34 (0.25–0.48)	**<.0001**	0.62 (0.43–0.91)	**0.015**
South countries	1.12 (0.83–1.53)	0.46	1.44 (1.01–2.05)	**0.045**
Age ≥ 75 years	1.39 (1.03–1.87)	**.029**	1.04 (0.74–1.45)	0.82
Body mass index ≤25 kg/m^2^	1.41 (1.06–1.88)	**.019**	1.68 (1.22–2.31)	**0.002**
Hypertension	0.60 (0.44–0.80)	**.0006**	0.59 (0.43–0.83)	**0.002**
Diabetes	0.71 (0.54–0.94)	**.015**	0.60 (0.43–0.82)	**0.001**
Ischemic heart disease	1.32 (1.01–1.73)	**.04**	1.53 (1.13–2.08)	**0.006**
LVEF ≤30%	1.52 (1.17–1.98)	**.002**	1.18 (0.87–1.60)	0.28
Orthopnea	1.71 (1.29–2.27)	**.0002**	1.49 (1.08–2.06)	**0.015**
Systolic BP ≤110 mmHg	2.64 (1.91–3.65)	**<.0001**	2.76 (1.94–3.94)	**<0.0001**
eGFR <45 ml/min/1.73m^2^	1.70 (1.19–2.42)	**.003**	3.05 (2.10–4.43)	**<0.0001**

	Unspecified reasons		Symptoms or side effects	
Beta‐blocker model	OR (95% CI)	p value	OR (95% CI)	p value
European geographical area				
Central countries	(reference)		(reference)	
North countries	0.22 (0.15–0.32)	**<.0001**	0.38 (0.25–0.58)	**<.0001**
South countries	0.57 (0.39–0.84)	**.004**	0.63 (0.41–0.98)	**.039**
Hypertension	0.67 (0.50–0.89)	**.006**	0.86 (0.61–1.20)	0.36
Orthopnea	1.54 (1.13–2.10)	**.006**	2.01 (1.42–2.84)	**< .0001**
Heart rate ≤ 70 bpm	1.40 (1.04–1.89)	**.027**	1.84 (1.31–2.58)	**.0004**

*Note*: Patients who reached target doses are considered a reference group.

Bold values if *p*‐value < 0.05.

Abbreviations: ACEi, angiotensin converting enzyme inhibitor; ARB, angiotensin receptor blocker; CI, confidence interval; eGFR, estimated glomerular filtration ratio; LVEF, left ventricular ejection fraction; OR, odd ratio.

Regarding beta‐blockers with successful up‐titration as the reference group, patients from Central European centers, those with more severe congestion and those with lower heart rate were less likely to be up‐titrated to target doses (in both groups a) and b) that is, without and with reason provided). Table 2.

### 3.3 Adverse events of up‐titrating ACEi/ARB or beta‐blockers

At 9 months, hypotension, hyperkalemia (>5.0 and 5.5 mmoL/L) and renal impairment occurred in 3.8%, 14.9%, 4.3% and 8.9% of patients, respectively. For ACEi/ARB, after adjustment for potential confounders, hypotension, hyperkalemia and renal impairment occurred at similar rates among 3 up‐titration groups of patients (all adjusted p values > 0.10). Table 3. In patients with an age ≥ 75 years, female sex, a BMI≤25 kg/m^2^, a LVEF≤30% or an eGFR≤60 ml/min/1.73m^2^, there was no between‐group difference in adverse events (adjusted p values > 0.10 for all subgroups) (Supplementary Table 2).

**TABLE 3:** Comparison of adverse events associated with ACEi/ARB or beta‐blockers among patients not reaching target doses for unspecified reason, those not reaching target doses due to symptoms/side effects, and those reaching target doses

ACEi/ARB	Unspecified reasons	Symptoms or side effects	Target doses	Adjusted p value
Hypotension, *N* (%)(SBP < 90 mmHg)	31 (3.5%)	27 (6.5%)	7 (1.7%)	0.31
Renal impairment, *N* (%)(eGFR<30 ml/min/1.73m^2^)	54 (8.5%)	40 (13.4%)	17 (5.4%)	0.11
Hyperkalemia, *N* (%)(Potassium>5.0 mmoL/L)	102 (16.6%)	40 (13.7%)	39 (12.8%)	0.64
Hyperkalemia, *N* (%)(Potassium>5.5 mmoL/L)	23 (3.7%)	19 (6.5%)	10 (3.3%)	0.22
Beta‐blockers	Unspecified reasons	Symptoms or side effects	Target doses	Adjusted p value
Hypotension, *N* (%)(SBP < 90 mmHg)	48 (4.2%)	11 (3.3%)	6 (2.7%)	0.51
Bradycardia, *N* (%)(Heart rate < 50 bpm)	18 (1.6%)	3 (0.9%)	3 (1.3%)	0.48

*Note*: Comparisons among three groups were adjusted for covariates which were selected in multinomial logistic regression analyses for ACEi/ARB or beta‐blocker, respectively.

Abbreviations: ACEi, angiotensin converting enzyme inhibitor; ARB, angiotensin receptor blocker; SBP, systolic blood pressure; eGFR, estimated glomerular filtration rate.

For beta‐blockers, bradycardia and hypotension occurred in 1.5% and 3.8% of patients, respectively. After adjustment for potential confounders, bradycardia and hypotension occurred at similar rates among the three groups of patients (all adjusted p values > 0.10). Table 3. In patients with an age ≥ 75 years, a BMI≤25 kg/m^2^, a heart rate ≤ 70 bpm, NYHA of III/IV and impaired quality of life assessed by KCCQ score, there was no between‐group difference in the studied adverse effects (adjusted p values > .05 for all subgroups) (Supplementary Table 3).

During the following stabilization period for ACEi/ARB, 7.2% (*N* = 76) and 0.9% (*N* = 5) of patients in group (a) and (b) were successfully up‐titrated, respectively, whereas 9.8% (*N* = 46) of patients in group (c) did not maintain their target doses until 9 months. A sensitivity analysis excluding these patients showed similar incidence of adverse events among three groups (Supplementary Table 4). Similarly, for beta‐blockers, we excluded 5.6% (*N* = 78), 1.2% (*N* = 5) and 16.0% (*N* = 41) of patients in group (a), (b) and (c), and observed similar rates of adverse events among three groups (Supplementary Table 4).

## 4 DISCUSSION

In this prospective study of treatment optimization including a large number of patients with symptomatic HFrEF on suboptimal therapy, we show that patients in whom no reason was reported for not reaching target doses of ACEi/ARB and/or beta‐blockers had a similar risk profile to those not up‐titrated due to side effects. On the other hand, patients reaching target doses had generally better clinical status.

### 4.1 ACE‐inhibitor or ARB up‐titration

In the present analysis, and consistently with prior reports, patients with ischemic heart disease, those with more severe congestion, those with lower SBP and those with poorer renal function were less likely to initiate or reach target doses of ACEi/ARB.^8^, ^18^, ^24^, ^25^, ^26^, ^27^ Older age and lower EF were associated with poorer up‐titration only in the group of patients for whom no reason for no up‐titration tentative was provided. These findings may be due to the general perception of higher rate of adverse effects related to advanced disease, comorbidity burden, frailty or polypharmacy/risk of drug–drug interactions in elderly patients,^18^, ^20^, ^28^, ^29^ and suggest that clinicians introduce *priors* in their decisions which is consistent with the Bayes' theorem that integrates previous knowledge related to the conditions that may influence an event or intervention. The introduction of *priors* in human decisions has been seminally described elsewhere,^30^ and suggests that our experience may serve as an anchor on which we hold for decision making. In other words, applying to the current example, elderly patients with more comorbid conditions experience more side‐effects from treatments, especially at higher doses, and this is observed in daily practice and confirmed by data; hence, many clinicians may assume that all elderly/sick HF patients will experience side‐effects and, therefore, do not deserve to be up‐titrated. However, this clinical inertia may not hold in all cases, as we observed that patients with successful up‐titration of ACEi/ARB had similar rates of hypotension, hyperkalemia and renal impairment to those previously reported in clinical trials.^2^, ^3^, ^31^ In a report of the Effects of High‐dose versus Low‐dose Losartan on Clinical Outcomes in patients with Heart Failure (HEAAL) trial (*N* = 3846), patients assigned with the high‐dose losartan had low rates of side effects, but, numerically, these side effects were slightly higher compared with those with the low‐dose losartan.^2^, ^32^, ^33^ In the Comparative Effects of Low and High Doses of the Angiotensin‐Converting Enzyme Inhibitor, Lisinopril, on Morbidity and Mortality in Chronic Heart Failure (ATLAS) trial (*N* = 3164), comparing between high‐ and low‐dose groups, relatively small difference in mean SBP (<5 mmHg) and similar incidence rate of major increases in creatinine (>1 mg/dl) were observed.^3^ A report of the Clinical Outcome with Enalapril in symptomatic chronic Heart Failure: a dose comparison (NETWORK) trial (*N* = 1532) comparing high‐, medium‐ and low‐dose of enalapril showed similar rates of renal impairment and hyperkalemia across three dose strata.^31^ Importantly, it should be noted that there was no dose‐dependent difference in incidence rate of adverse events leading to drug discontinuation in these clinical trials. Generally, certain subgroups, such as the elderly, those with impaired renal function, those with low BMI and/or diabetes are reported to be at higher risk of adverse effects associated with ACEi/ARB,^18^, ^34^ and among subgroups with these high‐risk profiles, higher doses of ACEi/ARB may increase a risk of adverse effects.^35^, ^36^ However, in the present study, we found similar proportion of adverse events between older patients who reached target doses and those who did not, suggesting that this high‐risk subgroup should generally be treated with guideline‐directed medical therapy. Body size may be also the dominant reason for prescribing lower doses. A recent registry data showed that patients with a lower BMI were less likely to be up‐titrated.^37^ However, incidence of adverse events did not differ across BMI categories in the present analysis. In addition, we observed similar adverse events in patients with low blood pressure and/or those with poor renal function. Importantly, treatment up‐titration may thrive higher absolute net benefit in these high‐risk subgroups.^38^, ^39^, ^40^

### 4.2 Beta‐blocker up‐titration

Only about 10% of patients reached target doses of beta‐blockers. Regardless of reason for not up‐titrating, and consistently with previous findings, patients who had lower heart rate and those with more severe congestion were less likely to be up‐titrated to target doses.^8^, ^22^, ^41^ As for ACEi/ARBs, this finding may be explained by clinicians' *prior* concern about the safety of beta‐blockers in patients with lower heart rate and congestion. As for ACEi/ARBs, in the present analysis, we observed low rates of adverse events (e.g., bradycardia and hypotension) associated with the prospective up‐titration of beta‐blockers.^4^, ^5^ In the Carvedilol produces Dose‐related Improvements in Left Ventricular Function and Survival in subjects with chronic Heart Failure (MOCHA) trial comparing high‐, medium‐, and low‐dose carvedilol in 345 patients with chronic HF, higher doses of carvedilol were associated with higher incidence of bradycardia, but without compromising the benefit of high‐dose carvedilol.^4^ It should be noted, however, that in MOCHA, the majority of patients (>90%) received digitalis, which may increase dose‐dependent incidence rate of bradycardia in combination with beta‐blockers.^42^ A more recent report showed no association between beta‐blocker dose and bradycardia, which is in line with our findings.^5^ Also, data from large‐scale registries showed that older age, lower heart rate, decreased quality of life and/or female sex were associated with increased risk of adverse events.^8^, ^22^ However, in our report, there was no significant difference in rate of adverse events in each high‐risk subgroup, irrespective of the reason of not up‐titrating to target doses. These findings suggest again that physicians may be concerned about the likelihood of drug intolerance in high‐risk HF patients, consequently triggering therapeutic inertia.

### 4.3 Clinical implications

Several reports suggest that heart failure medications are under‐prescribed and often not optimally up‐titrated, especially in subgroups with high risk of adverse events, such as the elderly, those with multiple concomitant treatments, high comorbidity burden, low blood pressure or impaired renal function.^13^, ^19^, ^38^, ^39^, ^40^, ^43^, ^44^ In addition, recent registry data showed that in approximately 20% of patients, provider‐related issues such as provider inertia and aversion were underlying reason for dose decrease or discontinuation of ACE inhibitor/ARB or beta‐blockers.^8^

Our data show that sub‐optimal HF therapy up‐titration is not solely related to objective clinical presentations (e.g., low blood pressure, hyperkalemia, or poor renal function). Many clinicians may have their decisions influenced by a prior belief that, in high‐risk patients, side‐effects will occur in the short‐term which may be perceived as more important than the potential long‐term benefits. However, from these observational findings, one cannot determine if the adverse event pattern among these patients would be better or worse than that of patients not up‐titrating due to objective reasons. Behavioral interventions, including formal training in risk management and decision‐making, and established algorithm might mitigate physician inertia and risk aversion.^45^, ^46^, ^47^

## 5 LIMITATIONS

Although BIOSTAT‐CHF prospectively enrolled HFrEF patients who had no optimal doses of HF medications, with the aim of therapy optimization, our study has several limitations. This study relies on a post‐hoc analysis, hence the causality cannot be inferred. The BIOSTAT‐CHF study was designed to address ACEi/ARB and beta‐blocker up‐titration, thus these data may not be suitable for accounting for mineralocorticoid receptor antagonist prescription or treatment dose. Although we tried to eliminate bias as much as possible using covariate adjustment, unmeasured potential confounders may remain. No up‐titration for unclear reason may be likely due to the short titration period, patient or physician compliance to the guidelines‐based recommendation. Although investigators were instructed to record all reasons for dose change per the protocol of BIOSTAT‐CHF, further specific reasons for not reaching target doses was lacking. Furthermore, types of health care providers (i.e., general practitioners or cardiologists) may influence our findings. We also may have not been able to exclude an attribution bias, that is, lower‐risk patients may receive higher doses of HF medications, and these patients are also less likely to experience adverse effects. We tried to study the high‐risk subgroups, but an attribution bias cannot be eliminated. Lastly, we had no available clinical follow‐up data at the end of the up‐titration period. However, a sensitivity analysis excluding patients who were changed their treatment doses during the stabilization period did not alter the interpretation of our results.

## 6 CONCLUSIONS

Patients in whom clinicians did not give a reason why up‐titration was missed were older and had more co‐morbidities. Patients in whom up‐titration was achieved did not have excess adverse events. However, from these observational findings, the pattern of subsequent adverse events among patients in whom up‐titration was missed cannot be determined.

## CONFLICT OF INTEREST

None.

## Supporting information


**Appendix S1**: Supporting Information

## Data Availability

The datasets generated during the current study are available from the corresponding author on reasonable request.
