# Supplementary material for: Perceived risk profile and treatment optimization in heart failure: an analysis from BIOlogy Study to TAilored Treatment in chronic heart failure
Source: Clin Cardiol. 2021 May 7;44(6):780–8. doi: 10.1002/clc.23576 (PMC8207977; doi:10.1002/clc.23576)
Supplement: Supplementary file 1 — Appendix S1: Supporting Information [file CLC-44-780-s001.docx]

**Supplementary table 1. Reasons about no up-titration of ACEi/ARB or beta-blockers due to symptom, side effects or non-cardiac organ dysfunction**

|  | **Symptoms**  **(e.g. cough)** | **Side effects**  **(e.g. hypotension)** | **Non-cardiac organ dysfunction**  **(e.g. renal dysfunction)** |
| --- | --- | --- | --- |
| **ACEi/ARB** | 186 (34.7%) | 277 (51.7%) | 73 (13.6%) |
|  |  |  |  |
|  | **Symptoms**  **(e.g. fatigue)** | **Side effects**  **(e.g. bradycardia)** | **Non-cardiac organ dysfunction**  **(e.g. COPD-related respiratory disorder)** |
| **Beta-blocker** | 201 (47.9%) | 204 (48.6%) | 15 (3.6%) |

**Supplementary table 2. Comparison of adverse events among patients not reaching target doses of ACEi/ARB for unspecified reasons, those not reaching target doses due to symptoms/side effects and those reaching target doses across high risk subgroups**

| ACE inhibitor or ARB model | Unspecified  reasons | Symptoms or  side effects | Target doses | Adjusted  p-value |
| --- | --- | --- | --- | --- |
| Age ≥75yrs |  |  |  |  |
| Hypotension (SBP<90 mmHg), N (%) | 8 (2.8 %) | 5 (4.5 %) | 4 (4.0 %) | 0.34 |
| Renal impairment (eGFR<30 ml/min/1.73m²), N (%) | 31 (13.8 %) | 16 (16.8 %) | 8 (9.3 %) | 0.69 |
| Hyperkalemia (Potassium>5.0 mmol/l), N (%) | 36 (16.7 %) | 14 (15.6 %) | 11 (13.1 %) | 0.72 |
| Male |  |  |  |  |
| Hypotension (SBP<90 mmHg), N (%) | 27 (4.1 %) | 21 (6.7 %) | 5 (1.6 %) | 0.26 |
| Renal impairment (eGFR<30 ml/min/1.73m²), N (%) | 35 (7.3 %) | 29 (13.1 %) | 11 (4.6 %) | 0.13 |
| Hyperkalemia (Potassium>5.0 mmol/l), N (%) | 81 (17.3 %) | 32 (14.7 %) | 30 (13.1 %) | 0.65 |
| Female |  |  |  |  |
| Hypotension (SBP<90 mmHg), N (%) | 4 (1.9 %) | 6 (5.8 %) | 2 (2.2 %) | 0.81 |
| Renal impairment (eGFR<30 ml/min/1.73m²), N (%) | 19 (12.3 %) | 11 (14.5 %) | 6 (7.9 %) | 0.58 |
| Hyperkalemia (Potassium>5.0 mmol/l), N (%) | 21 (14.3 %) | 8 (10.8 %) | 9 (11.8 %) | 0.89 |
| Body mass index ≤25kg/m² |  |  |  |  |
| Hypotension (SBP<90 mmHg), N (%) | 16 (5.6 %) | 9 (6.3 %) | 4 (4.1 %) | 0.80 |
| Renal impairment (eGFR<30 ml/min/1.73m²), N (%) | 19 (9.3 %) | 15 (13.8 %) | 5 (6.1 %) | 0.23 |
| Hyperkalemia (Potassium>5.0 mmol/l), N (%) | 30 (14.9 %) | 18 (17.0 %) | 8 (9.6 %) | 0.73 |
| Body mass index >25kg/m² |  |  |  |  |
| Hypotension (SBP<90 mmHg), N (%) | 15 (2.6 %) | 17 (6.3 %) | 3 (1.0 %) | 0.053 |
| Renal impairment (eGFR<30 ml/min/1.73m²), N (%) | 34 (7.9 %) | 25 (13.4 %) | 11 (4.8 %) | 0.34 |
| Hyperkalemia (Potassium>5.0 mmol/l), N (%) | 72 (17.4 %) | 21 (11.5 %) | 31 (14.1 %) | 0.44 |
| Diabetes mellitus |  |  |  |  |
| Hypotension (SBP<90 mmHg), N (%) | 4 (1.5 %) | 6 (5.4 %) | 0 (0.0 %) | **0.009** |
| Renal impairment (eGFR<30 ml/min/1.73m²), N (%) | 26 (13.3 %) | 17 (22.7 %) | 6 (5.9 %) | 0.12 |
| Hyperkalemia (Potassium>5.0 mmol/l), N (%) | 36 (18.8 %) | 16 (22.5 %) | 15 (15.3 %) | 0.87 |
| Left ventricular ejection fraction ≤30% |  |  |  |  |
| Hypotension (SBP<90 mmHg), N (%) | 22 (4.2 %) | 17 (7.0 %) | 3 (1.4 %) | 0.30 |
| Renal impairment (eGFR<30 ml/min/1.73m²), N (%) | 27 (7.5 %) | 18 (10.8 %) | 9 (5.6 %) | 0.69 |
| Hyperkalemia (Potassium>5.0 mmol/l), N (%) | 55 (15.4 %) | 17 (10.5 %) | 19 (11.7 %) | 0.30 |
| Systolic blood pressure (SBP) ≤120mmHg |  |  |  |  |
| Hypotension (SBP<90 mmHg), N (%) | 26 (5.2 %) | 24 (10.3 %) | 6 (4.2 %) | 0.39 |
| Renal impairment (eGFR<30 ml/min/1.73m²), N (%) | 28 (7.9 %) | 26 (15.6 %) | 4 (3.4 %) | **0.049** |
| Hyperkalemia (Potassium>5.0 mmol/l), N (%) | 61 (17.4 %) | 24 (14.5 %) | 13 (11.7 %) | 0.80 |
| eGFR ≤60 ml/min/1.73m² |  |  |  |  |
| Hypotension (SBP<90 mmHg), N (%) | 12 (3.1 %) | 15 (8.2 %) | 4 (2.9 %) | 0.31 |
| Renal impairment (eGFR<30 ml/min/1.73m²), N (%) | 49 (15.8 %) | 35 (24.8 %) | 12 (10.4 %) | 0.19 |
| Hyperkalemia (Potassium>5.0 mmol/l), N (%) | 56 (18.7 %) | 24 (17.1 %) | 19 (17.0 %) | 0.88 |

Comparisons among three groups were adjusted for covariates which were selected in multinomial logistic regression analysis.

ACEi, angiotensin converting enzyme inhibitor; ARB, angiotensin receptor blocker; eGFR, estimated glomerular filtration rate.

**Supplementary table 3. Comparison of adverse events among patients not reaching target doses of beta-blockers for unspecified reasons, those not reaching target doses due to symptoms/side effects and those reaching target doses across high risk subgroups**

| Beta-blocker model | Unspecified  reason | Symptoms or  side effects | Target doses | Adjusted  p-value |
| --- | --- | --- | --- | --- |
| Age ≥75yrs |  |  |  |  |
| Hypotension (SBP<90 mmHg), N (%) | 11 (3.4 %) | 5 (4.4 %) | 1 (1.6 %) | 0.41 |
| Bradycardia (Heart rate <50 bpm), N (%) | 6 (1.8 %) | 1 (0.9 %) | 1 (1.6 %) | 0.71 |
| Male |  |  |  |  |
| Hypotension (SBP<90 mmHg), N (%) | 39 (4.4 %) | 8 (3.2 %) | 6 (3.6 %) | 0.69 |
| Bradycardia (Heart rate <50 bpm), N (%) | 15 (1.7 %) | 2 (0.8 %) | 2 (1.2 %) | 0.33 |
| Female |  |  |  |  |
| Hypotension (SBP<90 mmHg), N (%) | 9 (3.4 %) | 3 (3.5 %) | 0 (0 %) | 0.19 |
| Bradycardia (Heart rate <50 bpm), N (%) | 3 (1.1 %) | 1 (1.1 %) | 1 (1.8 %) | 0.95 |
| Body mass index ≤25kg/m² |  |  |  |  |
| Hypotension (SBP<90 mmHg), N (%) | 21 (5.8 %) | 7 (7.7 %) | 1 (1.4 %) | 0.20 |
| Bradycardia (Heart rate <50 bpm), N (%) | 7 (1.9 %) | 2 (2.2 %) | 1 (1.4 %) | 0.70 |
| Body mass index >25kg/m² |  |  |  |  |
| Hypotension (SBP<90 mmHg), N (%) | 26 (3.4 %) | 4 (1.6 %) | 5 (3.3 %) | 0.17 |
| Bradycardia (Heart rate <50 bpm), N (%) | 11 (1.4 %) | 1 (0.4 %) | 2 (1.3 %) | 0.37 |
| Sinus rhythm |  |  |  |  |
| Hypotension (SBP<90 mmHg), N (%) | 19 (3.4 %) | 3 (1.8 %) | 1 (1.1 %) | 0.23 |
| Bradycardia (Heart rate <50 bpm), N (%) | 7 (1.2 %) | 1 (0.6 %) | 1 (1.1 %) | 0.73 |
| Atrial fibrillation |  |  |  |  |
| Hypotension (SBP<90 mmHg), N (%) | 17 (5.1 %) | 3 (3.4 %) | 3 (3.7 %) | 0.88 |
| Bradycardia (Heart rate <50 bpm), N (%) | 9 (2.7 %) | 1 (1.1 %) | 1 (1.2 %) | 0.32 |
| Systolic blood pressure (SBP) ≤120mmHg |  |  |  |  |
| Hypotension (SBP<90 mmHg), N (%) | 41 (6.7 %) | 10 (6.2 %) | 5 (4.5 %) | 0.81 |
| Bradycardia (Heart rate <50 bpm), N (%) | 10 (1.6 %) | 1 (0.6 %) | 1 (0.9 %) | 0.52 |
| Heart rate ≤70 bpm |  |  |  |  |
| Hypotension (SBP<90 mmHg), N (%) | 16 (3.8 %) | 4 (2.8 %) | 4 (6.2 %) | 0.30 |
| Bradycardia (Heart rate <50 bpm), N (%) | 7 (1.7 %) | 0 (0 %) | 1 (1.5 %) | 0.06 |
| NYHA III/IV |  |  |  |  |
| Hypotension (SBP<90 mmHg), N (%) | 33 (5.0 %) | 7 (3.5 %) | 2 (1.7 %) | 0.25 |
| Bradycardia (Heart rate <50 bpm), N (%) | 13 (2.0 %) | 1 (0.5 %) | 1 (0.8 %) | 0.09 |
| KCCQ ≤60 |  |  |  |  |
| Hypotension (SBP<90 mmHg), N (%) | 20 (5.1 %) | 3 (3.4 %) | 2 (3.0 %) | 0.71 |
| Bradycardia (Heart rate <50 bpm), N (%) | 9 (2.3 %) | 0 (0 %) | 1 (1.5 %) | 0.16 |

Comparisons among three groups were adjusted for covariates which were selected in multinomial logistic regression analysis.

SBP, systolic blood pressure; NYHA, New York Heart Association; KCCQ, Kansas City Cardiomyopathy Questionnaire.

**Supplementary table 4. Sensitivity analysis excluding patients who were changed their prescribed doses during the stabilization period**

| ACEi/ARB | Unspecified reasons | Symptoms or  side effects | Target doses | Adjusted  p-value |
| --- | --- | --- | --- | --- |
| Hypotension, N (%) (SBP<90 mmHg) | 31 (3.9 %) | 26 (6.3 %) | 6 (1.6 %) | 0.45 |
| Renal impairment, N (%) (eGFR<30 ml/min/1.73m²) | 53 (9.1 %) | 40 (13.6 %) | 13 (4.6 %) | 0.15 |
| Hyperkalemia, N (%) (Potassium>5.0 mmol/l) | 95 (16.8 %) | 40 (13.9 %) | 38 (13.9 %) | 0.79 |
| Hyperkalemia, N (%) (Potassium>5.5 mmol/l) | 20 (3.5 %) | 19 (6.6 %) | 10 (3.7 %) | 0.20 |
| Beta-blocker | **Unspecified reasons** | **Symptoms or**  **side effects** | **Target doses** | **Adjusted**  **p-value** |
| Hypotension, N (%) (SBP<90 mmHg) | 46 (4.3 %) | 11 (3.3 %) | 5 (2.6 %) | 0.49 |
| Bradycardia, N (%) (Heart rate <50 bpm) | 18 (1.7 %) | 3 (0.9 %) | 3 (1.6 %) | 0.44 |

Comparisons among three groups were adjusted for covariates which were selected in multinomial logistic regression analyses for ACEi/ARB or beta-blocker, respectively.

ACEi, angiotensin converting enzyme inhibitor; ARB, angiotensin receptor blocker; SBP, systolic blood pressure; eGFR, estimated glomerular filtration rate.
